# Surveillance epidemiology of heatstroke in China: when numerators are denominators

**DOI:** 10.1016/j.lanwpc.2025.101751

**Published:** 2025-11-21

**Authors:** John S. Ji

**Affiliations:** Vanke School of Public Health, Tsinghua University, Beijing, 100084, China

In the age of big data, surveillance systems capture vast amounts of information, but the bottleneck lies in interpretation for timely public health action. Heatstroke is a textbook example of surveillance epidemiology of a climate-sensitive illness. A recent analysis based on China CDC's *Heatstroke Cases Reporting System* reported 86,406 heat-related illness cases from 2010 to 2023.^1^ From this single case pool, public health professionals derived several key metrics: incidence density and case-fatality rate (CFR).

First, the 86,406 cases serve as the *numerator* for calculating the incidence rate, estimated at 44.83 per 10 million person-years. The mortality rate is estimated at 0.89 deaths per 10 million person-years (using deaths derived from these cases). Second, the same cases form the *denominator* *to calculate the* overall CFR at 1.98%, rising to 6.19% among the 32% of patients classified as severe. Surveillance data on heatstroke are valuable, but they invite questions.

## Is it real?

The population-based denominator for incidence and mortality rates (cases or deaths ÷ person-years) is not static but actually dynamic in China, where migration, tourism, aging, and seasonal labor continuously reshape the at-risk population. The case-based denominator for CFR (deaths ÷ cases) can be skewed or biased upward when mild or community-managed cases go unreported, shrinking the denominator and misrepresenting the full clinical spectrum. Both measures also fluctuate due to healthcare-seeking behavior, operations (holidays, staffing), detection (awareness campaigns), system changes (reporting rules, insurance), and variable diagnostic criteria.

## Is it meaningful?

Assessing whether rising high-temperature days represent a genuine public health threat requires more than counting cases, it demands thoughtful interpretation of surveillance data. *Incidence* *rate* measures how often heatstroke occurs in the population, guiding prevention targets such as early warnings, cooling centers for the public, and occupational protection for outdoor workers. *CFR* reflects lethality, informing resource allocation for public health response and emergency medical services.

Globally, heat-related mortality averages 489,000 deaths annually (2000–2019), with 45% occurring in Asia.^2^ By contrast, China's clinically confirmed heatstroke surveillance system reports a mortality rate of only 0.89 per 10 million person-years, a value that is orders of magnitude lower than model-based heat-attributable mortality estimates. The difference largely reflects different denominators and case definitions: the Chinese registry captures only diagnosed heatstroke cases, while global estimates include all excess deaths statistically attributable to high temperatures. For comparison, in the United States, extreme heat contributes to 750–1300 deaths annually,^3^ and occupational heat fatality rates reach 0.22 per million workers.^4^ These higher rates indicate broader attribution methods capture the full health impact of heat exposure.

## Improving interpretability of surveillance data

To make sense of such apparent differences, surveillance data must be interpreted through the lens of denominator uncertainty, a common source of bias in rate-based epidemiology. As Sander Greenland notes, static person-time approximations can distort incidence estimates, particularly where migration and demographic shifts are rapid.^5^ He advocates Monte Carlo sensitivity analysis and multiple-bias modeling to quantify how denominator uncertainty affects results.^6^

In the present study, population denominators derived from the 2010 or 2020 censuses implicitly assume linear or stable growth, which may not hold in regions with strong seasonal population fluctuations. In such contexts, incidence can be misestimated by up to 50% if dynamic denominators are ignored.^7^ Proxies for population mobility, such as nighttime-light imagery or mobile–phone activity, could improve estimation of transient at-risk groups.

While denominator uncertainty affects rate accuracy, model choice also matters for count data. The use of negative binomial regression is appropriate, as it accounts for overdispersion and enhances the reliability of incidence modeling for rare outcomes like heatstroke.^1^

Not every analysis requires complex bias modeling, however, as Rothman and colleagues remind us in *Modern Epidemiology*, mid-interval population estimates are generally sufficient for rare outcomes when population change is slow and linear.^8^ This simplicity promotes transparency and reproducibility. Standardization techniques, such as age-adjusted rates, and sensitivity checks comparing census versus annual data, often suffice.

Beyond static surveillance, time-series approaches, such as distributed lag non-linear models (DLNMs), can capture both immediate and delayed temperature effects on heatstroke, accounting for nonlinear exposure–response patterns.^9^ Embedding these models in quasi-experimental frameworks—such as interrupted time series or difference-in-differences—could help evaluate interventions like heat-warning systems or emergency preparedness policies.

## Take away

Disease surveillance registries are difficult to establish and maintain. They require uniform reporting standards, trained personnel, and consistent field investigations across many jurisdictions. The teams who built and sustained these systems need to be commended, because only through such registries can we gain critical insights in climate health risks. In China, data collected over a decade reveal a clear relationship between high-temperature days and heatstroke incidence.

All health data is a mosaic, its patterns shifting with the distance of our gaze, up close, a scatter of imperfect tiles: biases, uncertainty, fragments of truth, but step back one fact remains: at least 86,406 people in China suffered heatstroke between 2010 and 2023, with one third of cases classified as severe. This number, likely conservative, underscores a pressing reality: high-temperature days pose a real threat to human health. Surveillance data, is not just a pixel of information, but a call to action: adapt before the heat claims more.

## Contributors

John S. Ji conceived and conceptualised the comment, conducted the writing of the manuscript.

## Declaration of interests

John S. Ji is a Senior Technical Officer for Environment, Health and Epidemiology at the WHO Asia–Pacific Centre for Environment and Health in the Western Pacific Region.
